# A Rare Primary Adrenal Malignancy Manifesting as a Hemorrhagic Mass: Case Report and Literature Review

**DOI:** 10.1155/crie/1260707

**Published:** 2026-04-08

**Authors:** Salem Al Ghaithi, Ibrahim Alali, Ali Al Reesi, Ali Almadhani

**Affiliations:** ^1^ Endocrinology and Diabetes Unit, Internal Medicine Department, Sohar Hospital, Ministry of Health, Sohar, Oman, moh.gov.om; ^2^ Hematology Unit, Internal Medicine Department, Sohar Hospital, Ministry of Health, Sohar, Oman, moh.gov.om

**Keywords:** adrenal glands, adrenal hemorrhage, EBV-associated lymphoma, primary natural killer/T cell lymphoma, SMILE chemotherapy

## Abstract

Primary adrenal extranodal natural killer/T‐cell lymphoma (ENKTL) is an exceptionally rare, aggressive malignancy with fewer than 10 cases worldwide. We describe a 55‐year‐old man with diabetes mellitus and benign prostatic hyperplasia who presented with progressive left‐sided abdominal pain and a 17‐kg unintentional weight loss over 4 months. Imaging showed a 4.9‐cm left adrenal mass with hemorrhagic features, initially presumed to be spontaneous adrenal hemorrhage. Endocrine evaluation excluded functional adrenal tumors, and surgical management required conversion from laparoscopic adrenalectomy to open resection due to superior mesenteric vein (SMV) involvement. Histopathological and immunophenotypic confirmed non‐nasal ENKTL (CD3+, CD56+, granzyme B+, perforin+, and Epstein–Barr virus‐encoded RNA [EBER]+). Postoperative ^18^F‐FDG PET/computed tomography (CT) demonstrated residual disease; steroid, methotrexate, ifosfamide, L‐asparaginase, and etoposide (SMILE) chemotherapy achieved a marked complete metabolic response after six cycles—one of the most significant documented responses in primary adrenal NK/T‐cell lymphoma. This case highlights diagnostic challenges of primary adrenal lymphoma (PAL), the need for histopathological confirmation in atypical masses, and suspicion for hematological malignancies with systemic symptoms and adrenal lesions.

## 1. Introduction

The natural killer (NK)/T cell lymphomas are aggressive lymphoproliferative disorders derived from activated NK cells or cytotoxic T cells. The World Health Organization classifies NK–cell malignancies into extranodal NK/T‐cell lymphoma (ENKTL; nasal and non‐nasal), aggressive NK‐cell leukemia and chronic lymphoproliferative disorder of NK cells [1]. Primary adrenal lymphoma (PAL) comprises <1% of extranodal lymphomas, with diffuse large B‐cell lymphoma (DLBCL) being the most common subtype [2]. ENKTL, is an aggressive, Epstein–Barr virus (EBV)‐associated malignancy nasal ENKTL predominantly affects the upper aerodigestive tract (especially nasal cavity) and is more common in Asian populations, while non‐nasal (extranasal) ENKTL arises at other extranodal sites, often presents at advanced stages, and has poorer prognosis [3]. Primary adrenal involvement is exceptionally rare, likely due to limited adrenal lymphoid tissue and ENKTL tropism for epithelial/mucosal sites. Like other ENKTLs, primary adrenal ENKTL, non‐nasal type shows characteristic vascular destruction, necrosis, and strong EBV association. It often presents diagnostic challenges due to its nonspecific clinical and radiological features, which frequently mimic benign conditions such as adrenal hemorrhage or adenoma. Immunophenotypically, the tumor cells express CD2, CD56, and cytotoxic markers, and are EBV‐encoded RNA (EBER)‐positive, confirming EBV infection [1, 4].

Therapeutic approaches for primary adrenal non‐nasal ENKTL borrow from extranasal protocols. Anthracycline‐based regimens are ineffective due to MDR1‐mediated resistance [5]. Instead, treatment relies on asparaginase‐based non‐anthracycline combinations—most notably the steroid, methotrexate, ifosfamide, L‐asparaginase, and etoposide (SMILE) protocol—and radiotherapy for localized disease [3]. In advanced, relapsed, or refractory cases, outcomes remain poor, and novel agents such as programmed death‐1 (PD‐1)/PD‐ligand 1 (PD‐L1) inhibitors (e.g., pembrolizumab and sintilimab) are under investigation given frequent PD‐L1 overexpression. It has shown promising objective response rates (ORRs; 40%–75%) in relapsed/refractory ENKTL, especially combined with chemotherapy, with trials exploring frontline and resistant settings [5]. Early diagnosis and combined chemoradiotherapy offer the best chance for improved survival, but overall prognosis remains guarded given the aggressive nature of this lymphoma subtype. We report a case of primary adrenal NK/T‐cell lymphoma (non‐nasal type) initially misdiagnosed as spontaneous hemorrhage, highlighting the need for histopathological confirmation and multidisciplinary care for accurate diagnosis and effective treatment.

## 2. Case Presentation

A 55‐year‐old male with a history of uncontrolled type 2 diabetes mellitus managed with insulin therapy and benign prostatic hyperplasia on tamsulosin. He presented with left‐sided abdominal pain and unintentional weight loss of 17 kg over 4 months. He denied fever, night sweats, or anorexia. Physical examination revealed normal vital signs and no lymphadenopathy; no clinical features of Cushing’s syndrome, virilization, or androgen excess. The abdomen was soft with splenomegaly (4 cm below the left costal margin) but no hepatomegaly. Initial laboratory studies revealed seemingly reassuring results, with normal hemoglobin levels (15.0 g/dL) and adequate platelet counts (322 × 10^3^/µL). However, careful scrutiny revealed concerning findings that later proved diagnostically significant. The absolute neutrophil count was reduced to 0.7 × 10^3^/µL, suggesting possible bone marrow involvement or systemic disease. Initial peripheral blood smear showed large platelets, hypochromic microcytic red cells, occasional elliptocytes, reactive lymphocytes, and rare atypical lymphocytes with bipolar cytoplasm. A repeat smear came normal, highlighting transient nature—possibly early subclinical bone marrow involvement or paraneoplastic effects consistent with the episodic nature of cytopenias in some ENKTL cases without overt leukemic phase.

**Figure 1:**
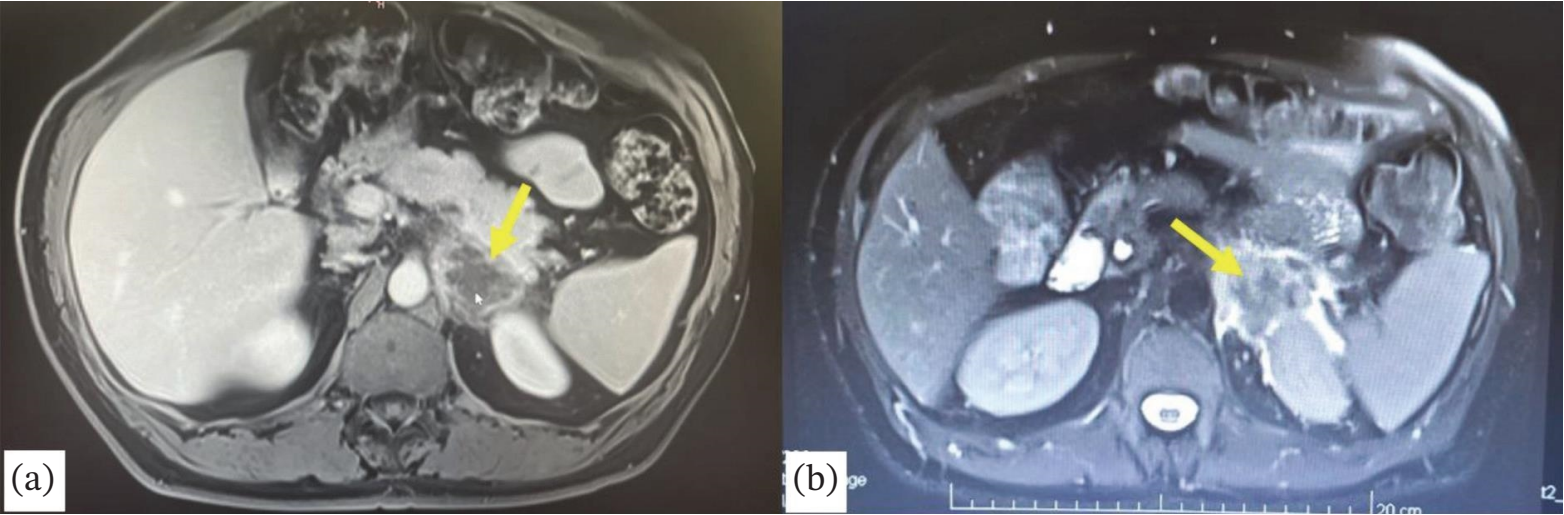
(a) Axial T1 weighted image shows the left adrenal mass (yellow arrow head) and (b) T2 weighted image shows the left adrenal mass (yellow arrow head).

Initial abdominal ultrasonography confirmed splenomegaly (142 mm) with homogeneous echotexture and no focal lesions or adrenal abnormality. Contrast‐enhanced PAN CT then identified a 3.1 cm × 4.4 cm × 4.9 cm ill‐defined soft tissue mass at the upper pole of the left kidney, with extensive perirenal fat stranding obscuring the left adrenal gland. The lesion abutted the splenic and short gastric veins and showed nodular thickening of the left diaphragmatic crus, suggesting adrenal neoplasm. Magnetic resonance imaging (MRI) of the adrenal region demonstrated classic hemorrhagic features—T2 hypointensity, iso‐ to low‐intensity signal on T1, and peripheral heterogeneous enhancement amid surrounding fat stranding—strongly indicating adrenal hemorrhage, as shown in Figure 1.

A comprehensive hormonal workup ruled out functional adrenal tumors. The 1 mg dexamethasone suppression test showed cortisol 38 nmol/L (<50 normal), excluding Cushing’s syndrome. Plasma metanephrines (0.1 nmol/L; normal 0–0.5) and noremetanephrines (0.33 nmol/L; normal 0–1) were normal, excluding pheochromocytoma. Aldosterone 392 pmol/L, renin 149 mIU/L, and aldosterone–renin ratio 2.6, excluding primary aldosteronism. Adrenal androgen measurement was not performed due to absent virilizing features. These results confirmed a nonfunctional adrenal lesion, making hemorrhage or benign nonfunctioning adenoma the leading possibilities.

Given the large adrenal mass with hemorrhagic features, unexplained weight loss, and splenomegaly (initially thought reactive/congestive from presumed hemorrhage; later recognized as extranodal involvement)—the decision was made to proceed with surgical exploration for definitive tissue diagnosis. The surgical procedure, initially planned as a straightforward laparoscopic left adrenalectomy, rapidly became one of our institution’s most challenging cases. Initial laparoscopic exploration revealed a massive, complex tumor with extensive dense adhesions that far exceeded expectations from preoperative imaging.

Intraoperatively, the tumor encased and invaded the superior mesenteric vein (SMV), requiring urgent vascular consultation and complex venous reconstruction. Extensive invasion, need for clear margins, and anatomical distortion necessitated conversion to open surgery. R2 resection (macroscopic residual disease) was achieved due to vascular encasement. The most difficult step was careful tumor dissection from the SMV to preserve vascular continuity. The patient remained hemodynamically stable throughout. The resected 5 cm heterogeneous specimen showed hemorrhage and necrosis, consistent with an aggressive lesion.

**Figure 2:**
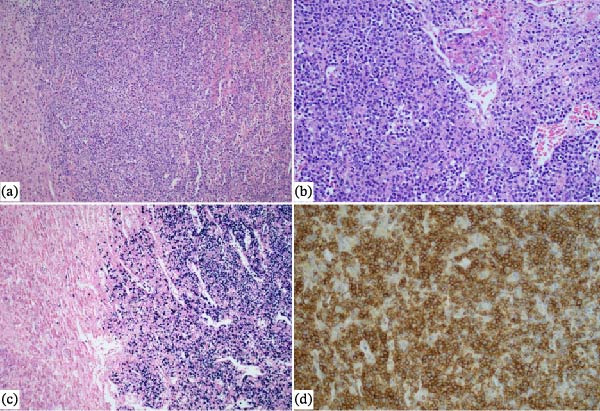
(a) Normal adrenal tissue on left side, with dense infiltration by sheets of atypical medium sized lymphoid cells (HE x100). (b) The lymphoid cells show angioinvasion with angiodestruction and necrosis (HE x200). (c) Atypical lymphoid cells are CD56 positive. (d) Atypical lymphoid cells are EBER positive.

Histopathologic examination revealed adrenal tissue with diffuse infiltration by atypical medium‐sized lymphoid cells with extensive tumor necrosis. Angioinvasion and angiocentric destruction were present. Immunohistochemistry confirmed the diagnosis, as the atypical cells were positive for CD3, CD56, Granzyme B, perforin, and EBER. Histopathologic findings shown in Figure 2.

This immunophenotypic profile, combined with the morphological features, established the definitive diagnosis of E, non‐nasal type.

EBER positivity confirmed EBV association, consistent with NK/T‐cell lymphoma biology and its therapeutic implications. Postoperative PET–CT (performed shortly after surgery) revealed residual metabolically active tissue at the adrenal site (4.7 cm × 3.6 cm, SUV max 9.1) and FDG‐avid focus at the splenic inferior pole, consistent with localized extranodal disease (stage IE). SMILE chemotherapy was initiated as first‐line therapy given CHOP resistance. After six cycles of SMILE, end‐of‐treatment ^18^F‐FDG PET/CT demonstrated size and metabolic stability of the residual surgical bed lesion (3.4 cm × 3.1 cm), with mild FDG uptake (SUVmax 3.0; previously 3.7) and no other FDG‐avid lesions. Interpreted as Deauville score 3, indicating complete metabolic response without active residual or distant disease. Post‐chemotherapy adrenal MRI confirmed a 4 cm hematoma with no viable residual tumor, supporting treatment success and absence of active malignancy. These findings represent one of the most dramatic responses to SMILE therapy documented in the literature for primary adrenal NK/T‐cell lymphoma.

## 3. Discussion

PAL is a rare, with fewer than 200 cases reported in the literature. Rashidi and Fisher [2] conducted a systematic review of 187 PAL cases, identifying DLBCL as the predominant histology (78%), followed by other B‐cell lymphomas (15%) and T‐cell lymphomas (7%). NK/T‐cell lymphoma is exceptionally rare in the adrenal gland, with most reported cases involving secondary rather than primary involvement. Although disseminated ENKL involving adrenal gland is not uncommon, only six cases of primary adrenal NK/T‐cell lymphoma—with EBER positivity and expression of cytotoxic molecules, both considered mandatory for diagnosis—have been reported in the literature [1, 6].

The condition is more frequently reported in Asian populations, particularly in China and Korea, due to higher prevalence of EBV‐associated lymphomas in these regions [3]. The median age at diagnosis is approximately 30–40 years, younger than that of other typical PAL subtypes such as DLBCL (median age ~60 years) [6]. There is a male predominance (male‐to‐female ratio ~2:1), consistent with typical NK/T‐cell lymphoma epidemiology. Differential diagnoses include metastatic disease, primary adrenal malignancies such as adrenocortical carcinoma, and benign disease like adenoma, phaeochromocytoma, or tuberculosis [7].

The clinicopathological features of these six cases, along with the our case, are summarized in Table 1. Unlike most prior cases (5/6 bilateral), our patient had unilateral left adrenal involvement, similar to only one previous report. B symptoms were present (as in 4/6 prior cases), and splenomegaly was noted without adrenal insufficiency—consistent most reported cases. Treatment with SMILE yielded a marked complete metabolic response after six cycles, representing one of the most significant responses documented. All cases exhibited bulky lesions (diameter ≥50 mm). Half of the cases showed multisite involvement. Immunophenotypically, all but one case exhibited an NK‐cell phenotype (CD56‐positive), and all demonstrated a high Ki‐67 index. One report describes long‐term survival after aggressive treatment, including high‐dose therapy and autologous hematopoietic stem cell transplantation. Two cases were diagnosed as ENKL postmortem.

**Table 1:** Review of published cases of primary adrenal extranodal NK/T‐cell lymphoma and comparison with the current case.

Reference	Case 1 [8]	Case 2 [9]	Case 3 [10]	Case 4 [11]	Case 5 [12]	Case 6 [13]	Case 7 (our)
Age/sex	40/F	79/F	67/M	35/M	17/M	37/M	55/M
B symptoms	No	No	No	Yes	Yes	Yes	Yes
Adrenal insufficiency	No	Yes	No	No	No	No	No
Laterality (max tumor diameter mm)	Bilateral (L 75, R 31)	Bilateral (L 55, R 57)	Bilateral (size N/ A)	Left (50)	Bilateral (L 50, R 48)	Bilateral (L 138, R 101)	Left (50)
Other lesions	Soft tissue	None	CSF, bone marrow	None	Liver, lung, bone marrow, spleen	Nasal cavity, pancreas, paravertebra	Spleen
Diagnostic method	Surgical resection	Autopsy	CT‐guided biopsy	Surgical resection	Autopsy	CT‐guided biopsy	Surgical resection
EBV DNA (blood)	N/A	N/A	N/A	N/A	Detected	Detected	N/A
Immunophenotype (CD2, CD3, CD4, CD8, CD30, CD56, GzB, TIA‐1)	+, +, −, −, −, +, +, N/A	N/A, +, −, −, −, −, −, +	+, −, −, −, N/A, +, +, +	dim, dim N/A, N/A, N/A, +, +, +	N/A, −, N/A, N/A, N/A, +, +, +	+, +, −, −, −, +, +, +	N/A, +, N/A, N/A, N/A, +, +
EBER‐ISH	+	+	+	+	+	+	+
Ki‐67 (%)	70	77	N/A	75	N/A	95	N/A
Therapy	GLIDE, HDT + ASCT	None	Dexamethasone	Hyper CVAD/MA, CHOP	None	SMILE, Sib/PBSCT	SMILE (6 cycles)
Outcome	Survival >1 year	Early death	Early	Death at 3 months	Early death	CNS relapse, death at 10 months	Complete metabolic response after 6 cycles; under surveillance

*Note*: CHOP, cyclophosphamide, doxorubicin, vincristine, prednisolone; HDT + ASCT, high‐dose chemotherapy with autologous hematopoietic stem cell transplantation; MA, methotrexate, cytarabine; Sib/PBSCT, HLA‐identical sibling donor peripheral blood stem cell transplantation; SMILE, dexamethasone, methotrexate, ifosfamide, L‐asparaginase, etoposide.

Abbreviations: BM, bone marrow; CSF, cerebrospinal fluid; CNS, central nervous system; DEX, dexamethasone; EBER, EBV‐encoded RNA; GLIDE, gemcitabine, L‐asparaginase, ifosfamide, dexamethasone, etoposide; GzB, granzyme B; hyper‐CVAD, hyperfractionated cyclophosphamide, vincristine, doxorubicin, dexamethasone; Ki‐67 LI, Ki‐67 labeling index; n/a, not available; sIL‐2R, soluble interleukin‐2 receptor.

Radiologically, PAL typically presents as a large, heterogeneous adrenal mass with necrosis or hemorrhage, resembling pheochromocytoma, adrenocortical carcinoma, or hematoma. Computed tomography (CT) and MRI are critical for identifying adrenal masses but lack specificity, necessitating histopathological confirmation. NK/T‐cell lymphoma is characterized by angiocentric and angiodestructive growth, with immunohistochemical markers including CD3, CD56, granzyme B, perforin, and EBER [1].

Therapeutic approaches for PAL vary based on histology and disease extent. Surgical resection is often performed for localized disease or diagnostic purposes, followed by chemotherapy [5]. The SMILE regimen has emerged as a cornerstone for NK/T‐cell lymphoma due to its efficacy in EBV‐associated malignancies [5]. Yamaguchi and Miyazaki [5] reported a 5‐year overall survival rate of 47% in patients with advanced‐stage NK/T‐cell lymphoma treated with SMILE, highlighting the importance of early and aggressive intervention. Radiotherapy is frequently employed for localized or residual disease, particularly in NK/T‐cell lymphoma, which is radiosensitive. Prognosis in ENKTL remains guarded, depending on detectable EBV DNA (poorer outcome), high Ki‐67 (>70%), advanced stage, and early response to asparaginase‐based therapy (e.g., SMILE). In our case, complete metabolic response after six SMILE cycles, and MRI showing no viable residual tumor are highly encouraging signs. Elevated LDH is a recognized poor prognostic factor; its measurement aids risk stratification in suspected cases. Maintain high suspicion for hematological malignancies, including rare ENKTL, in atypical adrenal masses with hemorrhagic features, constitutional symptoms, and negative functional workup, as imaging often mimics benign hemorrhage. Histopathological confirmation is essential. Asparaginase‐based regimens (e.g., SMILE) are preferred over anthracyclines due to inherent resistance. Multidisciplinary input from endocrinology, hematology, surgery, and pathology optimizes outcomes.

## 4. Conclusions

In conclusion, we report a rare case of primary ENKTL of the adrenal gland, presenting as a spontaneous hemorrhagic mass. The patient achieved complete metabolic response (Deauville score 3) following six cycles of SMILE chemotherapy, with no evidence of residual viable tumor on end‐of‐treatment ^18^F‐FDG PET/CT or dedicated adrenal MRI (showing only postsurgical hematoma). This case underscores the critical importance of histopathological confirmation in atypical adrenal masses, especially with constitutional symptoms and negative functional evaluation, and highlights the efficacy of asparaginase‐based regimens such as SMILE in this aggressive malignancy. Early multidisciplinary management involving endocrinology, hematology, surgery, radiology, and pathology optimizes diagnostic accuracy and therapeutic outcomes in such exceptionally rare entities.

**Nomenclature**
18F‐FDG: 18F‐fluoro‐2‐deoxy‐D‐glucose
EBV: Epstein–Barr virus
MRI: Magnetic resonance imaging
NK/T cell: Natural killer/T cell
PD‐1/PD‐L1: Inhibitors is programmed cell death protein 1/programmed death‐ligand 1 inhibitors
PET/CT: Positron emission tomography/computed tomography
SMILE: Dexamethasone, methotrexate, ifosfamide, L‐asparaginase, and etoposide
SUVmax: Maximal standardized uptake value.

## Author Contributions

Salem Al Ghaithi involved in patient management, drafted the manuscript, reviewed final manuscript, and had full access to all of the data in this study, taking complete responsibility for the integrity of the data and the accuracy of the data analysis. Ali Almadhani was involved in patient management and reviewed the final manuscript. Ibrahim Alali and Ali Al Reesi have reviewed and revised the manuscript and accepted the final revision.

## Funding

This research received no external funding.

## Disclosure

All authors have read and approved the final version of the manuscript.

## Ethics Statement

Ethical approval was obtained from the Research and Ethical Review and Approval Committee, North Batinah Governorate, Ministry of Health, Oman (Ref: MoH/CSR/25/30094).

## Consent

Written informed consent was obtained from patient, and all identifying information has been anonymized.

## Conflicts of Interest

The authors declare no conflicts of interest.

## Data Availability

The data are available from the corresponding author. The data that support the findings of this study are available in the Supporting Information section of this article.
